# A Twinchild with Hydrocephalus, Requiring the Use of Perforator and Hook for Delivery

**Published:** 1887-04

**Authors:** J. McF. Gaston

**Affiliations:** Professor of Surgery in the Southern Medical College, Atlanta, Ga.


### A TWIN-CHILD WITH HYDROCEPHALUS, REQUIRING THE USE OF PERFORATOR AND HOOK FOR DELIVERY.

BY J. McF. GASTON, M. D.,
Professor of Surgery in the Southern Medical College, Atlanta, Ga.

Being called in consultation with Drs. Luther Stephens and W. B. Parks, on the morning of the 5th of March, 1887, in a case of labor, I got the following history of the patient : She had been delivered of a healthy child a few hours previously, and, upon examination subsequently, it was found that there remained much greater enlargement of the abdomen than would result from the placenta, and there was discovered by vaginal exploration a mass presenting at the superior strait which did not correspond to that of the secundines. On instituting a thorough manual examination, I was unable to determine the exact nature of the presentation, but supposed it to be the anterior presentation of a fœtal head, and recommended a delay of two or three hours, until uterine contraction should cause it to descend, when the forceps might be resorted to for delivery. I was summoned, accordingly, to come prepared for this procedure, and upon applying the instrument on the presenting mass and undertaking to make traction, it slipped off backwards on two occasions. Bringing the patient fully under the influence of chloroform, I then carried my hand up to the side of the presenting mass into the cavity of the womb, and diagnozed either a monstrosity or a hydrocephalic development, so that it was determined, with the concurrence of my colleagues, to use the perforator. Upon puncturing the protruding walls water flowed out freely, and, introducing a blunt hook into the opening, I had no difficulty in extracting the flaccid scalp and island-like ossific formations which had been separated by the enormous distension caused by the fluid. The body of a dead child followed, and soon afterwards the conjoined placentas came away, but there was a considerable blood-clot remaining in the womb, which required to be removed by the hand carried up into the cavity of this organ. After this, the womb contracted firmly, and there was no further hemorrhage. The patient has progressed favorably since, and presents (on the third day after the delivery) nothing unusual for the parturient state.

Had my colleagues and I availed ourselves of the anæsthetic at the outset, and explored the uterine cavity, as was done at last, much embarrassment would have been obviated ; and the practical lesson to be inculcated by this result is an early resort to chloroform and introduction of the hand into the womb.

